# Advanced maternal age affects their frozen-thawed embryo susceptibility to high oxygen environment

**DOI:** 10.1038/s41598-024-73894-8

**Published:** 2024-10-03

**Authors:** Dhakshanya Predheepan, Sujith Raj Salian, Shubhashree Uppangala, Guruprasad Kalthur, Borut Kovačič, Satish Kumar Adiga

**Affiliations:** 1 Centre of Excellence in Clinical Embryology, Department of Reproductive Science, Kasturba Medical College, Manipal, Manipal Academy of Higher Education, Manipal, 576104 India; 2 Division of Reproductive Genetics, Department of Reproductive Science, Kasturba Medical College, Manipal, Manipal Academy of Higher Education, Manipal, 576104 India; 3 Division of Reproductive Biology, Department of Reproductive Science, Kasturba Medical College, Manipal, Manipal Academy of Higher Education, Manipal, 576104 India; 4 University Medical Centre, Maribor, Slovenia

**Keywords:** Ovarian aging, Embryo development, Apoptosis, Vitrification, Thawing, Oxidative stress, Developmental biology, Endocrinology

## Abstract

Preimplantation embryos can experience stress from laboratory interventions and a sub-optimal culture environment. Though research has demonstrated advanced maternal age impairs oocyte quality, the response of embryos derived from such oocytes to vitrification-thawing and culture in a high oxygen (O_2_) environment in the assisted reproductive technology laboratory is unknown. Therefore, in this study, embryos produced by in vitro fertilization (IVF) using oocytes from two- and eight-month-old Swiss albino mice were vitrified and thawed during their 6–8 cell stage. and cultured at low oxygen (5%) tension (LOT) and high oxygen (20%) tension (HOT). Embryo development, apoptosis, inner cell mass (ICM) outgrowth proliferation ability in vitro and pluripotency were assessed. Embryos from advanced maternal age cultured at HOT showed reduced fertilizing ability (*p* < 0.05), poor survival post-thawing (*p* < 0.05), and increased apoptosis (*p* < 0.01) in comparison to sibling embryos cultured at LOT. Importantly, the extended culture of vitrified-thawed embryos from advanced maternal age led to a significant (*p* < 0.001) reduction in complete ICM outgrowth formation at HOT in comparison to the LOT environment. The findings of this study suggest that HOT is detrimental to embryos from advanced maternal age, and importantly, vitrified-thawed embryos are more susceptible to stress, which could have negative implications, especially during the peri-implantation developmental period.

**Supplementary Information:**

The online version contains supplementary material available at 10.1038/s41598-024-73894-8.

## Introduction

The maternal age is known to influence the success of in vitro fertilization (IVF) as the oocyte quality declines significantly with age^1^. Specifically, the maturation potential, chromosome segregation, mitochondrial function, and epigenetic modifications are affected in the aged oocytes^2,3^, which has the risk of aneuploidy, mitochondrial damage, and increased oxidative stress^4^. It is known that poor-quality gametes and embryos are at higher risk of experiencing stress during their development in an assisted reproductive technology (ART) laboratory.

Embryo cryopreservation technique is now an integral part of the ART program. Though cryopreservation by vitrification has been well-established, few studies have reported the negative influence of vitrification-thawing on embryo and fetal development^5,6^. The rate of apoptosis in vitrified-thawed embryos may have a potential impact on the subsequent embryo progression^7^. Consequently, studies have shown that vitrified-thawed embryos are more likely to have a higher incidence of apoptosis and DNA fragmentation^8^.

Oxygen (O_2_) environment during embryo culture is known to affect the pregnancy outcome in ART cycles^9^. Many ART clinics use incubators that provide ambient O_2_ levels for embryo culture due to low maintenance costs, which contrasts the relatively low O_2_ levels in the female reproductive tract that protect the embryo from oxidative stress^10^. The high O_2_ tension may generate oxidative stress and impair embryo development^11^. Human embryos are more susceptible to degenerative alterations when subjected to high O_2_ tension during thawing and culture, as compared to lower O_2_ tension^12^. A recent study has demonstrated how high O_2_ tension affects mouse embryo development and quality, especially when oocytes are derived from an endogenous stress environment caused by prepubertal hyperglycemia^13^. Since the cryopreservation process can affect the cell integrity, metabolism, and developmental potential of thawed embryos^14,15^, it is hypothesized that the embryos derived from the advanced maternal-aged individuals, when subjected to high O_2_ environmental culture, are at higher risk of experiencing stress during cryopreservation-thawing. To investigate this, this study was undertaken using Swiss albino mice as the experimental model to investigate the behaviour of in vitro fertilized embryos derived from the advanced maternal age when subjected to vitrification-thawing followed by the culture at varying O_2_ tension for an extended time period.

## Methods

### Animals and ethical clearance

Inbred Swiss albino mice were procured from the Central Animal Research Facility of the Manipal Academy of Higher Education. Six mice per cage were housed at controlled conditions of 23 ± 2 °C, 12 h light-dark cycle, 50 ± 5% humidity, and fed with a standard diet and water *ad libitum* were used in the study. Animal handling and research experiments were conducted as per the guidelines of the Institutional Animal Ethics Committee (IAEC/KMC/44/2021). Animals were allowed to acclimatize to the facility for 2 weeks before being put on trial/experiment. A minimum of three trials were performed for each outcome measure to ensure the reproducibility of the results. Cervical dislocation was used as an euthanasia procedure during the experiments.

### Experimental design

Two-month-old (*N* = 31) and eight-month-old (*N*= 126) female Swiss albino mice (hereafter referred to as 2M and 8M, respectively) were used in the experiments. These age groups represent the reproductive ages of ~ 20 and ~ 37 years in humans, respectively^16^. All investigations were conducted under identical culture conditions and by employing sibling oocytes for all the study groups. The study was conducted in accordance with the Animal Research: Reporting of In Vivo Experiment (ARRIVE) guidelines.

### Ovarian stimulation

Female mice were superovulated by injecting 5 IU of pregnant mare serum gonadotropin (PMSG, Sigma Aldrich, USA) intraperitoneally, followed by 10 IU of human chorionic gonadotropin (hCG, Eutrig-HP) 48 h later. Animals were weighed and sacrificed 13 h post-hCG injection.

### Assessment of ovarian histology and follicle count

A minimum of six ovaries were collected for each data point from animals across three study groups for the comparative assessment of ovarian histology. The ovaries were weighed and fixed in 10% formalin solution for histological analysis. Sections of 5 μm thickness were made and subjected to deparaffinization - rehydration prior to Haematoxylin and Eosin (H&E) staining for the qualitative assessment. Parallelly, the ovarian reserve was assessed quantitatively as described earlier^17^ by enzymatic isolation of follicles from the ovaries using 2 mg/mL trypsin-collagenase (Cat. No: RM713, Himedia, India and Cat. No: 9001-12-1; Gibco, USA, respectively) dissolved in Dulbecco’s Modified Eagle’s Medium (DMEM, Cat. No: D5648, Sigma Aldrich, USA) for 30 min. Isolated follicles were identified as primordial, primary, secondary, preantral, antral, and atretic follicles under light microscopy.

### In vitro fertilization and embryo culture

IVF was performed as described earlier^13^. Briefly, cauda epididymis was collected from Swiss albino male mice and teased to release spermatozoa in pre-warmed Earl’s balanced salt solution (EBSS, Cat. No: E2888, Sigma Aldrich, St. Louis, MO, USA) supplemented with 0.1% bovine serum albumin (BSA, Cat. No: A3311, Sigma Aldrich, St. Louis, MO, USA). The sperm suspension was centrifuged, and the pellet was overlaid with Potassium Simplex Optimization Medium (KSOM-AA, Cat. No: MR-107-D, Sigma Aldrich, St. Louis, MO, USA) supplemented with 0.1% BSA and incubated for swim-up for 45 min at 37 ^◦^C. The supernatant containing the motile spermatozoa was then collected, inseminated in a 4-well dish (Cat. No: 176740, Thermo Fisher Scientific, Waltham, MA, USA), and overlaid with oil. Then, oviducts were collected from Swiss albino female mice, and the oviduct was gently cut open to release the oocyte cumulus complexes (COCs) into EBSS supplemented with 0.1% BSA. COCs were transferred to the 4-well dish and co-incubated with washed spermatozoa (2 × 10^6^ spermatozoa/mL). COCs from one oviduct were transferred to 5% O_2_ or low oxygen tension (hereafter referred to as LOT) and 20% O_2_ or high oxygen tension (hereafter referred to as HOT). The gametes were co-incubated at 37 ^◦^C with 5% CO_2_ at two different O_2_ tensions (LOT and HOT) and assessed for fertilization at 10.5 h post-insemination (hpi) to understand the fertilizing ability in varying O_2_ environments. The oocytes were then washed, and they were either categorized as normally fertilized (containing two pronuclei and two polar bodies) or abnormally fertilized (with one or no pronuclei). Only normally fertilized oocytes (5 oocytes in 20 µL droplet) were transferred to fresh KSOM supplemented with 0.1% BSA and cultured at 37 ^◦^C with 5% CO_2_ at LOT and HOT conditions until 96 hpi. Embryos were assessed using an inverted microscope (IX73, Olympus, Tokyo, Japan) every 24 hpi (24, 48, 72, 96, and 108 hpi) to determine their developmental competence at LOT and HOT.

### Embryo vitrification and thawing

Embryo vitrification-thawing was performed using vitrification (Cat. No. VT601, Kitazato, Japan) and thawing (Cat. No. VT602, Kitazato, Japan) kits as per the manufacturer’s protocol with minor modifications. Briefly, embryos (6 to 8 cell stage at 56–60 hpi) were transferred to the equilibration solution (ES) and incubated for 10–12 min. Embryos were then exposed to vitrification solution (VS) for 1 min and loaded onto a cryolock (8–10 embryos/ Cryolock), then plunged into liquid nitrogen and stored for 1 h. These embryos were then thawed by immersing the cryolock in a prewarmed thawing solution (TS) and subsequently moved to a dilution solution (DS) and washing solution (WS) for 3 and 6 min, respectively. A survival check was performed by culturing embryos in KSOM-AA supplemented with 0.1% BSA after 3 h.

### TUNEL assay

Apoptosis in blastocysts was assessed by terminal deoxynucleotidyl transferase dUTP nick end labelling (TUNEL) assay (In situ Cell Death Detection Kit, TMR Red, Cat. No. 12156792910, Roche, Germany), which is based on the detection of single- and double-stranded DNA breaks that occur at the early stages of apoptosis. At 108 hpi, the blastocysts were rinsed thrice in Phosphate-Buffered Saline (PBS), transferred to a multi-well plate (Cat. No. 163118, Thermo Fisher Scientific, USA), and fixed overnight with 4% paraformaldehyde (w/v). The embryos were then rinsed three times in PBS, followed by one-hour permeabilization in permeabilization buffer (0.1% sodium citrate (w/v), 0.5% BSA (w/v) and 0.5% Triton X-100 (v/v) in PBS) at room temperature. The embryos were washed thrice in PBS containing 0.5% BSA and treated for 1 h at 37 °C with the TUNEL reaction mixture (9:1 ratio of TMR red labelling solution to enzyme solution). The embryos were washed and then counterstained with 4 µg/mL DAPI (4’,6’-diamino-2-phenylindole) to stain the nucleus to assess the total cell number. Embryos were then transferred to clean microscopic slides and examined under a fluorescence microscope (Carl Zeiss, Gottingen, Germany) to determine the total cell number and TUNEL-positive cells. The labelling index was calculated as the percentage of TUNEL-positive cells per blastocyst.

### ICM outgrowth assay

The ability of the blastocyst to attach and proliferate post-implantation in vitro was assessed using the ICM outgrowth assay. Blastocysts appearing morphologically normal with expanded blastocoel at 108 hpi were selected for ICM outgrowth assay, which was performed as described earlier^18^with minor modifications. Briefly, multi-well dishes (Cat. No. 07-200-92, Thermo Fisher Scientific, USA) were precoated with 0.1% gelatin (Sigma-Aldrich, Cat No. G1393) for 30 min at room temperature. Excess gelatin was removed, and the dishes were air-dried. Individual blastocysts on 108 hpi were transferred into each well containing 200 µL DMEM (Sigma-Aldrich, Cat. No. D5648) supplemented with 20% fetal bovine serum (FBS, Cat. No. 10270106, Gibco, USA), overlaid with oil and cultured until 204 hpi under LOT and HOT environments. Blastocyst attachment and proliferation were monitored at 156 hpi under an inverted phase contrast microscope (IX 73, Olympus, Japan). The ICM outgrowths (IO) at 204 hpi were classified based on the morphology and size of the outgrowths, as reported previously^19^: completely developed ICM outgrowth (CIO), large ICM outgrowth (LIO), small ICM outgrowth (SIO), and no ICM outgrowth (NIO). The images were captured using Olympus IX 73, Japan.

### Isolation of total RNA, cDNA synthesis, and gene expression analysis

Total RNA was extracted from a minimum of 60 blastocysts at 108 hpi or 10 ICM outgrowths (CIO and LIO at 204 hpi) per group per trial, as per the kit recommendations (15596018, Ambion Life Technologies, USA). Following the manufacturer’s instructions, total RNA was reverse-transcribed using random primers by a high-capacity cDNA RT kit (4368814, Applied Biosystems, USA). Using the SYBR green kit (Cat. No. RR420A, TAKARA BIO INDIA Pvt. Ltd, India) for the study of *Hif1α* expression, Taqman premix gene expression assay kit for the study of pluripotency markers *Oct4*, *Sox2*, *Nanog* and StepOneTM Real-Time PCR System, a quantitative polymerase chain reaction (qPCR) was performed (Thermo Fisher Scientific, USA). SYBR green assay (Sigma Aldrich, USA) for *Hif1α* (5’ CCTGCACTGAATCAAGAGGTTGC and 3’ CCATCAGAAGGACTTGCTGGCT) was used. The qPCR outcomes were standardized using actin (5’ CATTGCTGACAGGATGCAGAAGG and 3’ TGCTGGAAGGTGGACAGTGAGG) as a reference gene. The TaqMan assay (Thermo Fisher Scientific, USA) was employed to evaluate the expression of pluripotency markers *Oct4* (Mm0305391- 7Ig1), *Sox2* (Mm0305- 3810Is1), and *Nanog* (Mm020195- 50Is1). The qPCR outcomes were normalized using *Actb* (Mm00607- 939Is1) as the standard gene. To compare mRNA levels at the blastocyst stage, in vivo blastocysts were used as a reference sample. On the other hand, due to the proximity to physiological conditions, ICM outgrowths cultured at LOT were used as a reference sample to compare the mRNA levels in ICM outgrowths.

### Statistical analysis

Data are represented as mean ± standard error of the mean (SEM). Based on the distribution of data points, either One-way Analysis of Variance (ANOVA) or the Kruskal-Wallis test was used to analyze the data. The unpaired t-test or Mann–Whitney U test was used to analyze the data between the groups. All statistical tests were conducted using the GraphPad Instat software (Graphpad Inc., La Jolla, CA, USA) (https://www.graphpad.com/features). GraphPad Prism 8 (GraphPad Prism software, CA, USA) was used to provide the graphical representation of the data. The level of significance was set at 5% throughout the study. All the data provided are from a minimum of three independent trials.

## Results

### Age and the ovarian reserve

**Fig. 1:**
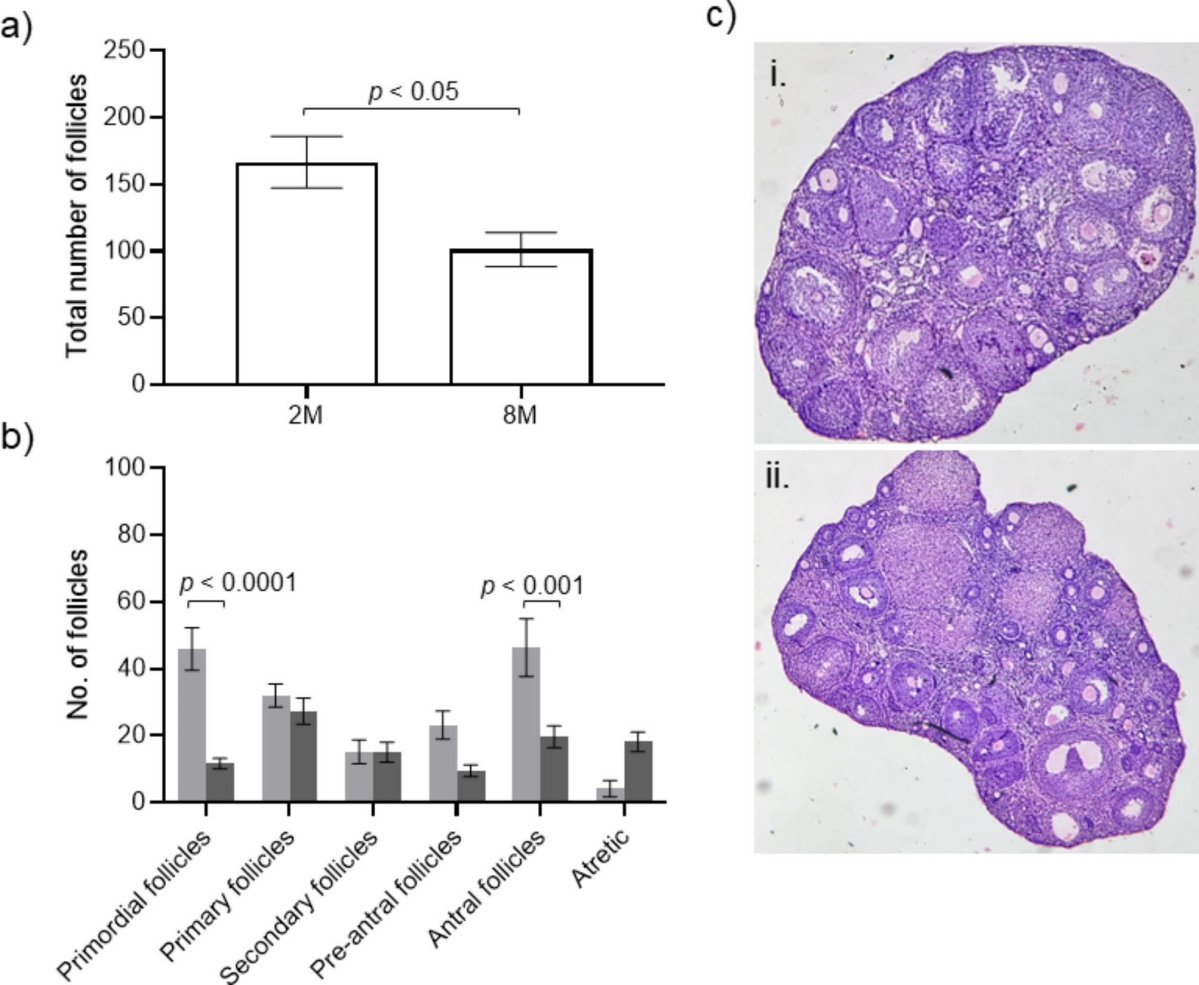
Influence of age on the ovarian reserve (a) Total follicle count per ovary *N* = 6 for 2M and 8M where ‘N’ indicates the number of animals included in the analysis. (b) Ovarian follicle count of mice aged 2M (light grey bar) *N* = 6 and 8M (dark grey bar) *N* = 6 respectively, where ‘N’ indicates the number of animals included in the analysis (c) Representative image of ovarian histology of 2M (i) 8M (ii) old mice (Magnification: 4X).

The total number of follicles in the ovary of the 8M age group was significantly lower than the 2M group (*p* < 0.05) (Fig. 1A). Specifically, a significant difference was found in the number of primordial (*p* < 0.0001) and antral (*p* < 0.001) follicles between 2M and 8M animals (Fig. 1B). This observation was supported histologically (Fig. 1C).

### Effect of varying O_2_ environment on vitrification-thawing survival of embryos

**Fig. 2:**
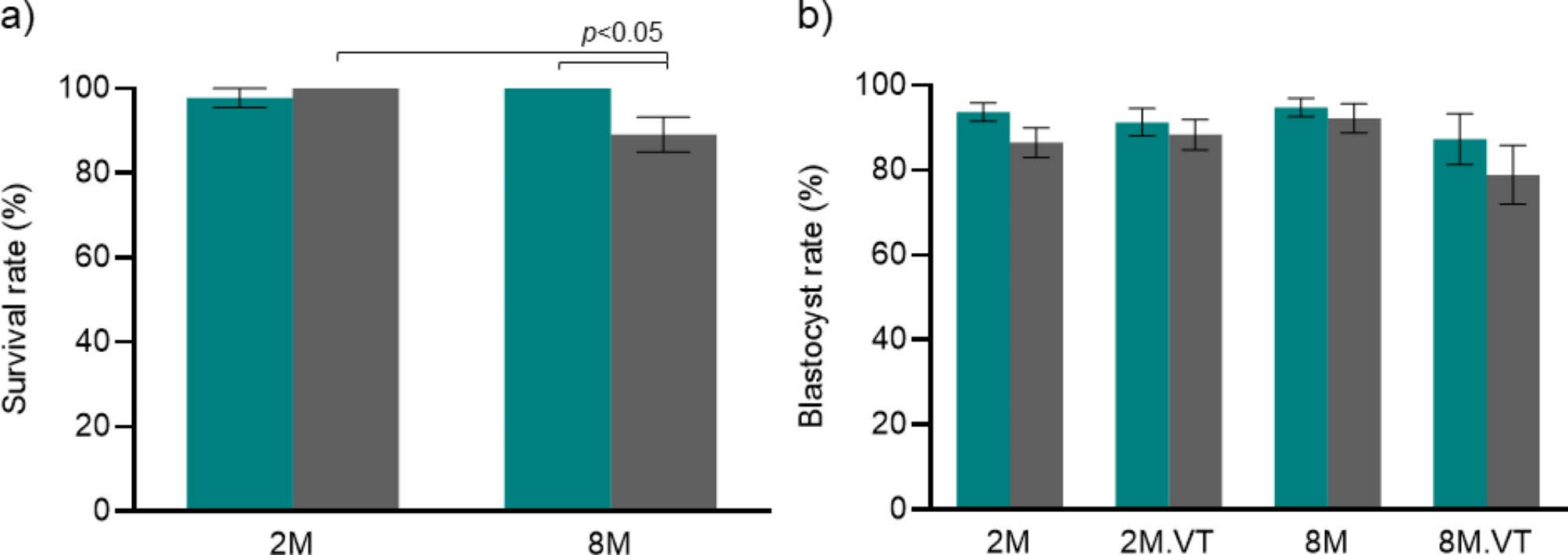
Influence of O_2_ environment on embryo survival and blastocyst formation of aged oocytes (a) Survival rate at LOT (Teal bar) *N* = 98, 173 for 2M and 8M, respectively, and HOT (Grey bar) *N* = 104, 236 for 2M and 8M, respectively, where ‘N’ indicates the number of 6–8 cell stage embryos vitrified-thawed (VT) (b) Blastocyst rate at LOT (Teal bar) *N* = 119, 96, 205, 173 for 2M, 2M.VT, 8M, and 8M.VT, respectively, and HOT (Grey bar) *N* = 114,104, 211,216 for 2M, 2M.VT, 8M, and 8M.VT, respectively, where ‘N’ indicates the number of 6–8 cell stage embryos (56 to 60 hpi) for the non-vitrified group and the number of 6–8 cell stage embryos (56 to 60 hpi) that survived post-thawing for the vitrified groups.

The fertilization rate was comparable when gametes from 2M animals were co-incubated at LOT (*N* = 333 metaphase II (MII) stage oocytes) and HOT (*N* = 308 MII oocytes) environments (83.89 ± 1.99 vs. 86.31 ± 2.38). Also, no significant variation in fertilization rate was observed between 2M (*N* = 333 MII oocytes) and 8M (*N* = 545 MII oocytes) groups at LOT (83.89 ± 1.99 vs. 89.08 ± 2.73). On the other hand, a significant (*p* < 0.05) increase in fertilization was observed in the 8M group when gametes were co-incubated at LOT (*N* = 545 MII oocytes) when compared to HOT (*N* = 625 MII oocytes) (89.08 ± 2.73 vs. 78.46 ± 3.13). Further, comparable post-thaw survival was observed in embryos cultured in the LOT environment prior to vitrification, irrespective of maternal age. In contrast, the 8M embryos exposed to HOT prior to vitrification demonstrated a significant (*p* < 0.05) reduction in the survival rate in comparison to LOT (Fig. 2A). However, the blastocyst forming potential and total cell number in the blastocysts were unaffected either by O_2_ environment or maternal age (Fig. 2B, Supplementary Fig. 1).

### Vitrified thawed embryos subjected to HOT had increased apoptosis

**Fig. 3:**
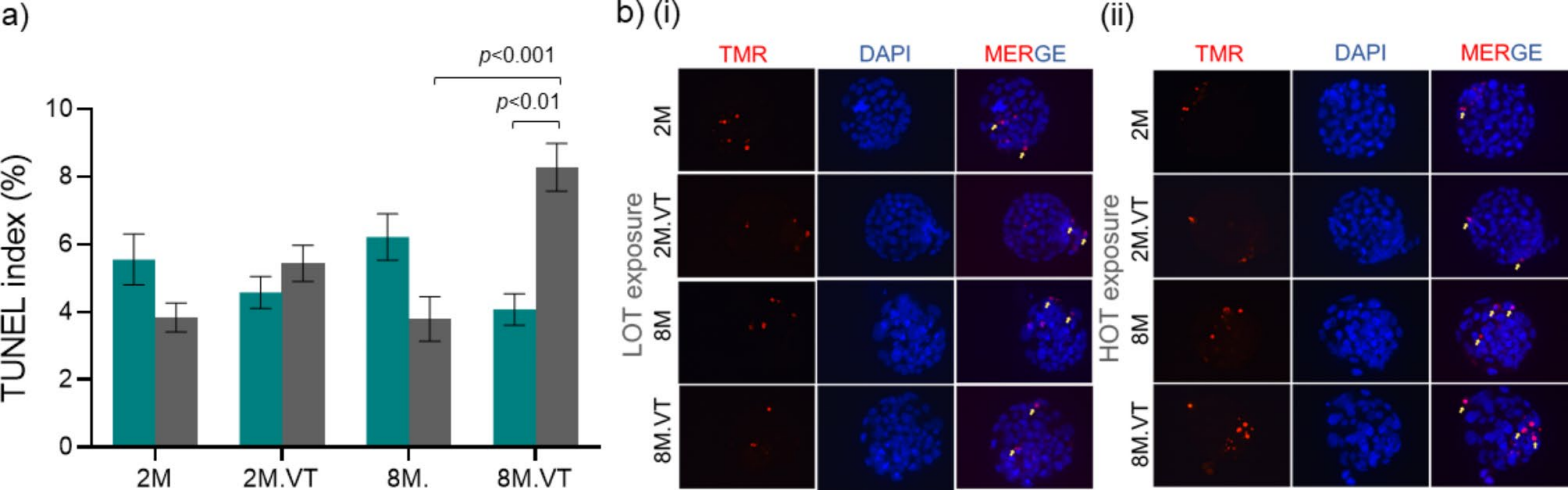
Effect of O_2_ environment on apoptosis (a) TUNEL index at LOT (Teal bar) *N* = 47, 54, 30, 34 for 2M, 2M.VT, 8M, and 8M.VT, respectively, and HOT (Grey bar) *N* = 69, 72, 31, 36 for 2M, 2M.VT, 8M, and 8M.VT, respectively, where ‘N’ indicates the number of blastocysts included in the analysis. (b) Representative images of TUNEL assay in blastocysts derived from LOT exposure (i) and HOT exposure (ii) (40X) (Scale bar: 20 μm). The yellow arrows indicate the TUNEL-positive cells.

The TUNEL labelling was performed to detect apoptotic cells in blastocysts. The TUNEL index was comparable between blastocysts derived from 2M to 8M maternal age groups when embryos were cultured at LOT and HOT. Interestingly, the TUNEL index was significantly (*p* < 0.01) higher in HOT-exposed 8M embryos subjected to vitrification-thawing in comparison to LOT-exposed, vitrified-thawed sibling embryos (Fig. 3A, B). Furthermore, the number of *Hif1α* transcripts in 8M vitrified-thawed embryos subjected to the HOT environment was marginally lower than the LOT-exposed sibling embryos but significantly (*p* < 0.01) lower than in vivo derived blastocysts (control) (Supplementary Fig. 2).

### Embryos exposed to HOT had reduced ICM expansion at 96 h of extended culture

**Fig. 4:**
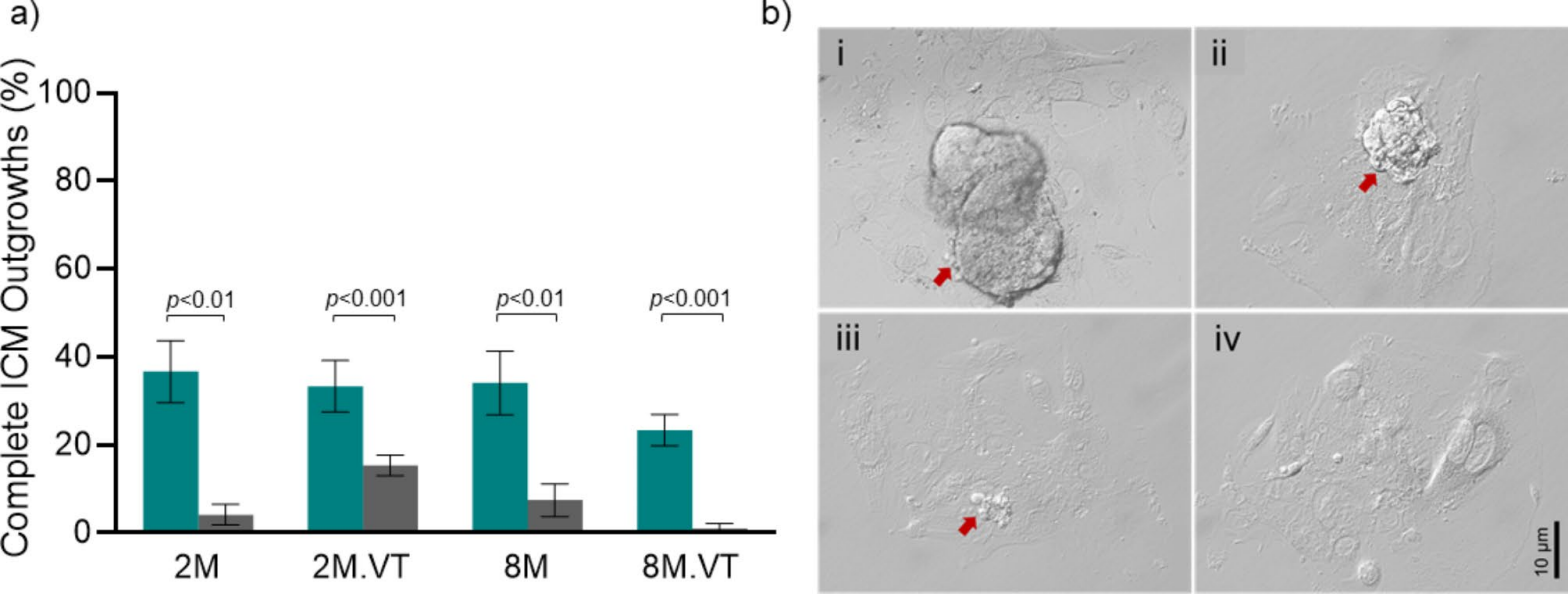
Influence of O_2_ environment on ICM outgrowth (a) Complete ICM outgrowth rate at LOT (Teal bar) *N* = 39, 31, 64, 71 for 2M, 2M.VT, 8M, and 8M.VT, respectively, and HOT (Grey bar) *N* = 41, 32, 84, 86 for 2M, 2M.VT, 8M, and 8M.VT, respectively, where ‘N’ indicates the number of blastocysts included in the analysis. (b) Representative phase-contrast images (i-iv) of ICM outgrowths at 204 hpi: completely developed ICM outgrowth (CIO), large ICM outgrowth (LIO), small ICM outgrowth (SIO), and no ICM outgrowth (NIO), respectively (40X). The red arrows indicate the ICM outgrowths (Scale bar: 10 μm).

The ICM expansion resulting in complete ICM outgrowths was assessed after 96 h of extended culture. Interestingly, a significant reduction in the complete ICM outgrowths was observed when embryos were cultured at HOT in comparison to the LOT environment (*p* < 0.001 - <0.01). Importantly, vitrification thawing of embryos from advanced age drastically affected the complete ICM outgrowth formation (Fig. 4A, B). Further, the number of *Hif1α*,* Oct4*, *Sox2*, and *Nanog* transcripts in ICM outgrowths were marginally reduced in HOT, though the differences were not statistically significant (Supplementary Fig. 3A-D).

## Discussion

This study investigated the susceptibility of embryos derived from the advanced maternal age to stressors such as high oxygen tension and vitrification-thawing. It is found that embryos from advanced maternal age, subjected to vitrification-thawing in conjunction with a high oxygen tension environment during in vitro culture, had increased apoptotic cells and poor ability of ICM proliferation in vitro compared to sibling embryos cultured at a low oxygen environment.

The aging of oocytes is marked by a series of molecular processes that progressively decline with age, leading to impairment in oocyte quality, fertilization, and development in experimental models^4,20,21^and in humans^22,23^. Studies also suggest that embryo apoptosis may be a contributing factor to impaired pregnancy outcomes with advancing age^24^. Earlier studies have demonstrated that oocytes from advanced maternal age are more prone to experience poor survival and increased spindle abnormalities post-vitrification-thawing compared to oocytes from younger animals^25^. It is possible that with advanced age, there is a decrease in the production of ATP and an increase in the reactive oxygen species (ROS) formation^26^, leading to an increase in oxidative stress thereby initiating oocyte aging^27^. The occurrence of oxidative stress in embryos developed in vivo is attributed to aging, and importantly, there can be additional iatrogenic stress during suboptimal in vitro environments such as O_2_ environment^28 ^and cryopreservation^12,15^. We have earlier observed the detrimental effect of the HOT environment when embryos from hyperglycemic mothers were cultured^13^.

The results from the present study clearly suggest that the HOT environment can significantly reduce the fertilization of oocytes from advanced maternal age compared to LOT. Moreover, the post-thaw survival rate of the embryos derived from aged oocytes was reduced when exposed to HOT, indicating that the embryos previously exposed to a high O_2_ environment can lose the survival ability post-vitrification-thawing. Although the blastocyst-forming ability and the total cell number in these embryos were comparable between varying O_2_ environments, the apoptosis was significantly higher in vitrified-thawed embryos from advanced maternal age subjected to HOT. Apoptosis eliminates potentially deleterious cells^29^due to oxidative damage and freeze-thaw process^14 ^and, therefore, serves as an indicator of the embryos’ quality during in vitro embryo culture^30^.

Oxidative stress can modify numerous crucial reactions that impact the process of embryonic development. Two recent studies have demonstrated that cryopreserved-thawed embryos, when subjected to HOT, experience altered metabolic activity^12^and impaired blastocyst formation^31^. The hypoxia-inducible factor (*Hif1α*) regulates genes involved in apoptosis and proliferation^32^, and it is known to protect against O_2 _tension-mediated apoptosis^33^. Our investigation revealed a considerable downregulation of the mRNA levels of *Hif1α* in blastocysts, particularly derived from advanced maternal age, cultured in a HOT environment, and subjected to vitrification-thawing. These results together indicate that subjecting embryos derived from advanced maternal age to vitrification-thawing and HOT culture poses a threat to genetic integrity at the peri-implantation phase of development.

Mouse pre-implantation embryos carrying DNA damage are morphologically identical to embryos with intact DNA^18,29,34^, and these embryos may continue to progress until the blastocyst stage as a result of a restricted checkpoint response during pre-implantation development^29^. Therefore, it is important to understand the in vitro proliferation ability of the inner cell mass of the blastocysts. The findings of our study indicate that HOT impairs the ICM expansion when compared to LOT, irrespective of maternal age, indicating a negative impact of HOT on embryonic development. However, vitrification thawing of advanced-age embryos has drastically reduced the ICM expansion at HOT, suggesting a detrimental effect during peri-implantation embryo development when cultured at HOT. *Hif1α* regulates pluripotency and proliferation of embryonic stem cells^35,36^. Our findings suggest that the expression of *Hif1α* and pluripotency markers *Oct4*, *Sox2*, and *Nanog* in top-quality ICM outgrowths showed a notable decline in the expression when non-vitrified embryos were subjected to HOT than the vitrified group. This phenomenon could be attributed to the fact that a considerable percentage of vitrified embryos exposed to HOT demonstrated deleterious consequences and ceased development at different phases and that only the successfully progressed ICM outgrowths were assessed for pluripotency.

The strength of this study is that it is the first experimental approach to examine the association between maternal age and susceptibility to cryopreservation, specifically in relation to varying O_2_ tension. Though this study was conducted in a rodent model due to ethical limitations, the observations possess a great translational value. However, these findings cannot be generalized to clinical practice at this juncture until further research is conducted specifically on human embryos. Furthermore, it is crucial to contemplate that in a clinical setting, individuals may be influenced by comorbidities in addition to advanced age; hence, the observations made here cannot be directly extrapolated.

## Conclusion

The preliminary results presented in this study indicate that embryos derived from advanced maternal age have increased cryo-susceptibility when exposed to the HOT environment *in vitro.* From the embryological perspective, this study highlights the importance of customizing embryo culture systems in ART clinics, particularly when maternal physiology impacts the ovarian environment.

## Electronic supplementary material

Below is the link to the electronic supplementary material.

Supplementary Material 1

Supplementary Material 2

Supplementary Material 3

## Data Availability

The datasets used and/or analysed during the current study are available from the corresponding author on reasonable request.

## Declarations

### Approval for animal experiments

This study was approved by the Institutional Animal Ethics Committee on 23 April 2021 by (IAEC/KMC/44/2021) the Manipal Academy of Higher Education.
