# Supplementary figures and images for: Advanced maternal age affects their frozen-thawed embryo susceptibility to high oxygen environment
Source: Sci Rep. 2024 Oct 3;14:23008. doi: 10.1038/s41598-024-73894-8 (PMC11450011; doi:10.1038/s41598-024-73894-8)

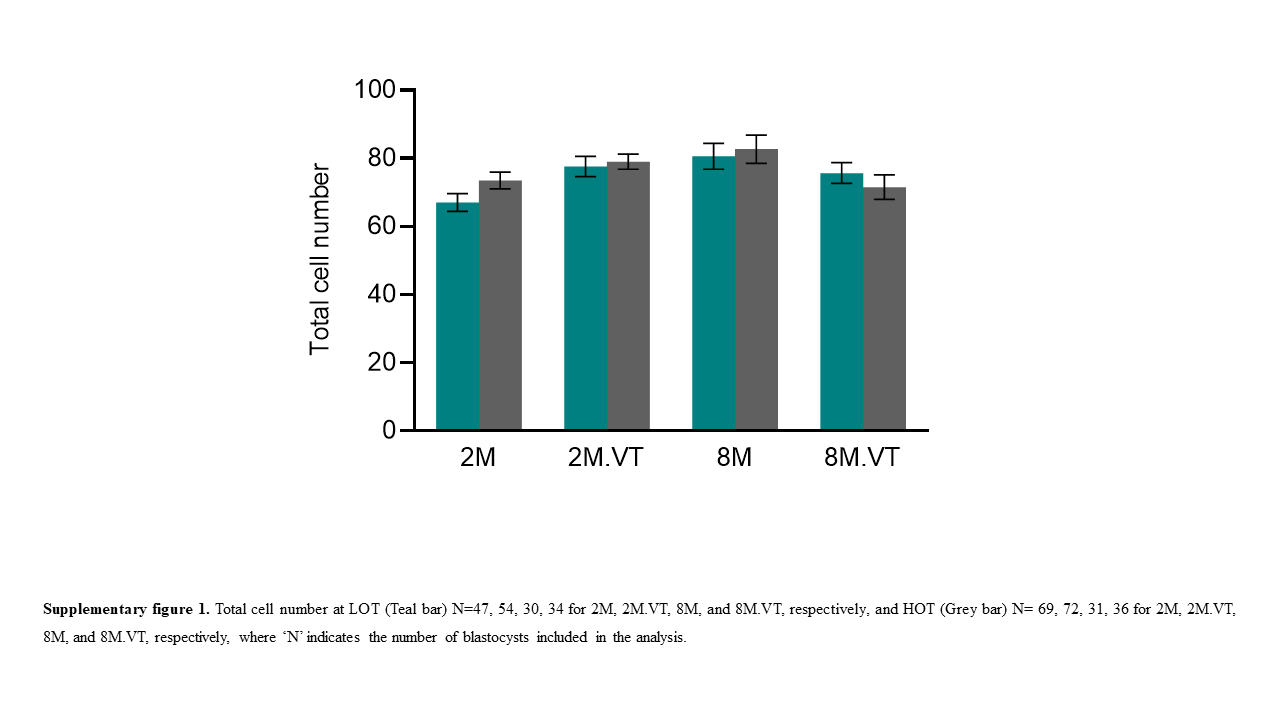

Supplement: Supplementary file 1 — Supplementary Material 1 [file 41598_2024_73894_MOESM1_ESM.tif]

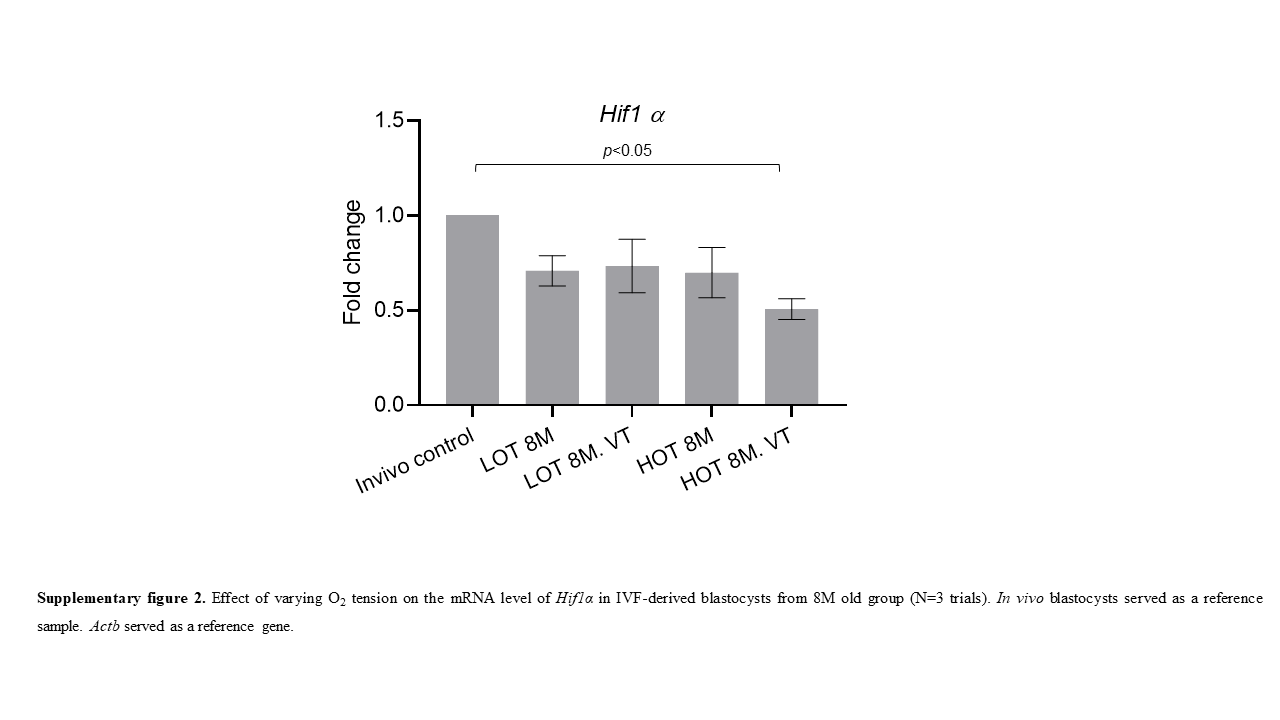

Supplement: Supplementary file 2 — Supplementary Material 2 [file 41598_2024_73894_MOESM2_ESM.tif]

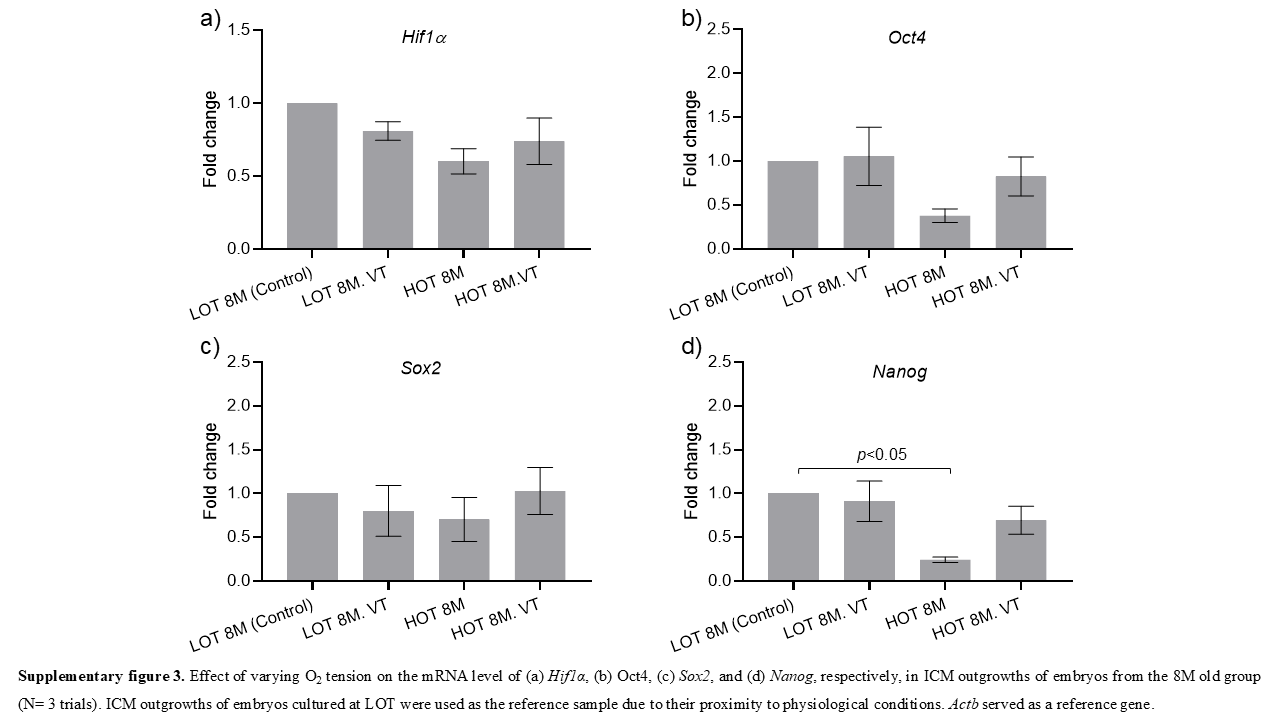

Supplement: Supplementary file 3 — Supplementary Material 3 [file 41598_2024_73894_MOESM3_ESM.tif]
